# Full-time Studies and Volunteering

**DOI:** 10.31729/jnma.4859

**Published:** 2020-02-29

**Authors:** Aliska Niroula, Shreedhar Prasad Acharya

**Affiliations:** 1 Kathmandu Medical College and Teaching Hospital, Sinamangal, Kathmandu, Nepal

Nepal Medical Association (NMA) is a professional organization of Medical and Dental Doctors in Nepal. It was established on 20th Falgun, 2007 BS (4th March 1951). It has been publishing an open access scientific publication, the Journal of Nepal Medical Association (JNMA) since 1963.^1^

Our volunteering began being a part of the editorial support team in the Journal of Nepal Medical Association. This has really built up our confidence to work as a team. During our days in Journal Office, a sudden knock of another opportunity struck our door. The Administrative Officer of Nepal Medical Association asked us about volunteering in the Election of Nepal Medical Association which was held from 17^th^ to 21^st^ Magh, 2076 and we accepted it. We also helped NMA during the pre-election procedure that needs to be done before going for the election. We think that was the work where we got the trust of NMA officers.

Time management is one of the important factors when we do multiple works and it is a difficult thing to do. Attending the lectures and going to the election site just after you finish the lectures with some biscuits in your hand. If we think of those days, we will surely ask ourselves a question, “how did we actually manage?” This taught us to manage our time which will surely help in our upcoming days. We, volunteers, have worked as a team during this election, we have done volunteering on a shift basis. As mentioned earlier, it’s a regular day volunteering so shifts were on the same day. Working as a team has really helped us in every challenge we faced during our volunteering period. Nothing can help us until we have the courage to perform it and there should always be someone who provides us that courage. During the time period of volunteering, the continuous guidance from our seniors was beneficial.

Working in an election was one of the best experiences that we had in medical school life. The election voting was done for 5 days and the voting system was well managed so that the voters can vote easily. Also, the system for voting was managed well to prevent double-voting with a single name. We performed digital entry, manual entry with the voters signature so as to prevent the double voting with a single name. We volunteers also helped the elder doctors to reach the secret room for filling up the ballot paper. Small tokens with serial numbers were provided to each voter so that the numbering of voters can be done later on and to cross-match it with the number of ballot papers inside the ballot box. We also didn’t allow the voters to take photos or videos inside the secret room as per the guideline of Election Commission. We checked the voters name in excel file which was prepared before the election and once the voters have voted, we ticked the name in the list as voted. CCTV surveillance was also present which shows the alertness of the election team. Identifcation, verifcation, providing the ballot paper, taking voters to the secret room and making them drop their paper in a systematic manner into the ballot box is actually the individual steps that we performed in this election. Besides these, there were so many legal procedures that our seniors performed to prevent future complications. For example, once the double paper was mistakenly provided to a single person but we could not identify the person to whom we gave it, in such condition our seniors called the candidate representative and wrote what actually happened and it was signed by the candidate representative and was sealed.

Usually, the vote-counting starts after the voting finishes. But, few doctors demanded a live telecast of the vote counting for the purpose of fairness. Many meetings were held to allow the vote counting. The vote counting was withheld for two days to ensure the system of vote counting among the representatives and the election committee. Candidates and candidate representatives were only allowed to seat in the vote-counting place. Counting six thousand votes and naming or registering 13 names for a single ballot paper continuously for 4 days with some breaks in between, this was actually what we did while counting votes. Google forms were created and the digital entry was done in the forms. We also counted the votes manually using the tally method. Basically, it was done for double checking the mistakes. Management of each ballot paper, showing the ballot paper to each of the representatives present inside the room and reciting it aloud, manual entry, digital entry and putting the bundles of ballot paper with signature into another box. During this phase, a lot of invalid ballot papers and invalid votes were seen. For example, two tick marks in two different candidate names. This way, we suggest to pre-inform the voters the way of casting votes in the upcoming election. We had minimal problems using google forms, although, it would be better if we use good software which makes the work more efficient. In an organization with above 10,000 members who are doctors by profession, it would be efficient if the vote casting system was made online. These are some of our suggestions and our learning curve from the election experience.

Through our continuous work, we gained trust from our seniors and a lot of opportunities have come to us like attending different medical conferences inside the country. Recently we attended the ORTHOCON 2020 where we learned a diverse range of things from academics to manage the program. Hence, we came to know that possibilities are endless. We are acknowledged to the NMA officers, the Election Committee and the JNMA team.

## Benefits of volunteering while studying

1. Work experience: We gain real-world skills which are very helpful in developing our career.
2. Teamwork: Working in a team deals with lots of things among which group dynamics is a thing which when maintained makes teamwork much more efficient and easy.
3. Ability to assess situations: We are exposed to a number of social and political issues while volunteering in various events and programs. We gain the ability to assess various situations in a compatible way.
4. Employability: If we can manage to volunteer and perform well even when no one is paying you, it makes a good impact on our commitment to do quality work.^2^
5. A good networking tool: Through volunteering, we are taking a step forward and opening ourselves up to people who are looking for candidates for jobs or other opportunities. Contacts are useful - you never know when you might need them.
6. A sense of purpose: Most of us feel a bit ‘lost’ at university which isn’t good for our mental health. Therefore, volunteering fills our time and gives us a sense of purpose. We practiced self-reflection and mindfulness which gave us a sense of purpose.
7. To find opportunities we never knew existed: By volunteering and grabbing one opportunity, we open ourselves up much more.^3^ As a volunteer, we gain the skills that make us to do more. Finally, we get to know possibilities are endless.

## Volunteering and studies

1. Planning semester in advance: Going through the syllabus and figuring out what we need to accomplish when. This makes us able to plan for our life bit by bit. It is important for maintaining a positive perspective on all areas of life.
2. Squeezing our time for studies: As the saying goes time and tides wait for no man, we got to know the value of each and every time and made each time valuable for our studies.

## Benefits of volunteering in abroad

Learning how to be independent, how to manage time and how to be responsible. These are skills that will shape our lives in the years to come, like an outline on how to live.^4^ Above all these are the primary traits to gain so that we can be able to live our lives in a new place far from home country. It also helps set you up for a future career.^2^

Volunteering in a field related to our career can help us gain experience in our area of interest and meet people in the field. Even if we are not planning on changing careers, volunteering gives us an opportunity to practice important skills used in the workplace - like teamwork, communication, problem-solving, planning and task management.

## The Right Organization Will Help You Make The Right Choices

As we are donating our valuable time, so it’s important that we enjoy and beneft from our volunteering.

To make sure that our volunteer position is a good fit:

1. Asking questions is a good way to make sure that the experience is right for our skills, our goals, and the time we want to spend.^5^
2. We should know what’s expected. You should be comfortable with the organization and understand the time commitment. Starting small is good so that we don’t over-commit ourselves at first. We should be flexible to change our focus if needed.
3. We should not be afraid to make a change. We should not force ourselves into a bad fit or feel compelled to stick with a volunteer role or program we dislike. Id something bad happens we should talk to the organization about changing our focus or look for a different organization that’s a better fit.
4. The best volunteer experience benefits both the volunteer and the organization- ultimately, it’s a win-win game!

These are the main things that made us able to work in the journal team and various other opportunities like election, election vote counting, and conferences.

## Conflicts of Interest:

None.
